# Exploring the Network between Adipocytokines and Inflammatory Response in SARS-CoV-2 Infection: A Scoping Review

**DOI:** 10.3390/nu15173806

**Published:** 2023-08-30

**Authors:** Ersilia Nigro, Vito D’Agnano, Gianluca Quarcio, Domenica Francesca Mariniello, Andrea Bianco, Aurora Daniele, Fabio Perrotta

**Affiliations:** 1 CEINGE-Biotecnologie Avanzate Scarl “Franco Salvatore”, Via G. Salvatore 486, 80145 Napoli, Italy; nigro@ceinge.unina.it (E.N.); aurora.daniele@unina.it (A.D.); 2 Dipartimento di Scienze e Tecnologie Ambientali, Biologiche e Farmaceutiche, Università della Campania “Luigi Vanvitelli”, Via Vivaldi 43, 81100 Caserta, Italy; 3 Department of Translational Medical Sciences, University of Campania L. Vanvitelli, 80138 Naples, Italy; vito.dagnano@studenti.unicampania.it (V.D.); gianluca.quarcio@studenti.unicampania.it (G.Q.); domenicafrancesca.mariniello@unicampania.it (D.F.M.); andrea.bianco@unicampania.it (A.B.); 4 Dipartimento di Medicina Molecolare e Biotecnologie Mediche, Università degli Studi di Napoli “Federico II”, 80055 Naples, Italy

**Keywords:** adipocytokines, SARS-CoV-2, COVID-19, adiponectin, leptin

## Abstract

Adipose tissue is actually regarded as an endocrine organ, rather than as an organ that merely stores energy. During the COVID-19 pandemic, obesity has undoubtedly emerged as one of the most important risk factors for disease severity and poor outcomes related to SARS-CoV-2 infection. The aberrant production of cytokine-like hormones, called adipokines, may contribute to alterations in metabolism, dysfunction in vascular endothelium and the creation of a state of general chronic inflammation. Moreover, chronic, low-grade inflammation linked to obesity predisposes the host to immunosuppression and excessive cytokine activation. In this respect, understanding the mechanisms that link obesity with the severity of SARS-CoV-2 infection could represent a real game changer in the development of new therapeutic strategies. Our review therefore examines the pathogenic mechanisms of SARS-CoV-2, the implications with visceral adipose tissue and the influences of the adipose tissue and its adipokines on the clinical behavior of COVID-19.

## 1. Introduction

**Figure 1:**
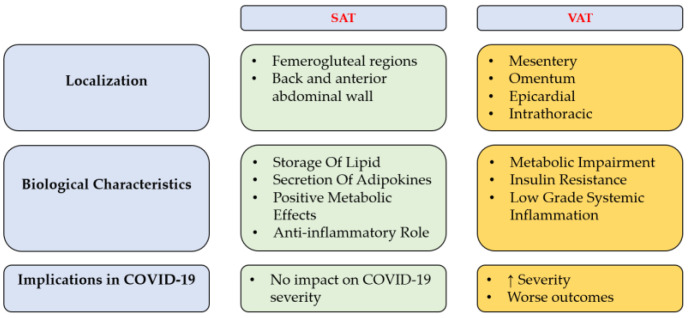
Difference between visceral adipose tissue (VAT) and subcutaneous adipose tissue (SAT).

Severe acute respiratory distress syndrome coronavirus 2 (SARS-CoV-2) is a novel coronavirus causing coronavirus disease 19 (COVID-19), which has spread worldwide, as the WHO declared a state of pandemic in March 2020. Clinical forms of the SARS-CoV-2 infection include asymptomatic, mild and severe forms, the last characterized by interstitial pneumonia and acute respiratory distress syndrome (ARDS). The general health status of the patient is directly connected to the risk of evolving into a severe clinical form, and different comorbidities, considered as risk factors, are related to worse outcomes of the disease. Among these comorbidities, obesity is a major risk factor for disease severity related to SARS-CoV-2 infection [1,2,3,4,5]. Although for a long time considered only an organ that stores energy, adipose tissue is currently considered a real endocrine organ [6]. The hypertrophy of adipocytes results in altered production of cytokine-like hormones, called adipokines, such as leptin and adiponectin, which contribute to metabolic alterations and dysfunction in the vascular endothelium and create a state of general chronic inflammation [7]. Adipocytes in different regions fulfill different physiological and pathophysiological functions. For example, it is important to highlight the difference between visceral adipose tissue (VAT) and subcutaneous adipose tissue (SAT): VAT is associated with metabolic impairment, such as insulin resistance and low grade systemic inflammation, while SAT has essential, positive functions, including storage of lipids and secretion of adipokines with positive metabolic effects and anti-inflammatory roles (Figure 1) [8,9,10,11]. Therefore, variations in visceral and/or subcutaneous adipose tissues may have significantly impact on the metabolic health state of a subject [7,12].

Since the start of the pandemic, epidemiological data have been analyzed worldwide, and many meta-analyses have been published, showing that patients affected by obesity are at greater risk for hospitalization and death [13,14,15,16,17]. Understanding the mechanisms that link obesity to the severity of SARS-CoV-2 infection could be the key to developing new therapeutic strategies and, more importantly, to preventing worsening of the disease in this group of patients.

## 2. Materials and Methods

A systematic literature search was conducted in Medline and the Cochrane Database, including articles published over the last three years (from 2020 to 2023). The review was conducted following the Preferred Reporting Items for Systematic Reviews and Meta-Analyses (PRISMA) checklist [18]. The following MeSH terms were used: SARS-CoV-2 OR COVID-19 AND Adipose tissue OR Adipocytokines OR Adiponectin OR Leptin OR Resistin OR apelin OR omentin1. Here, we report a narrative review of the abovementioned research.

## 3. SARS-CoV-2: Overview of Pathogenic Mechanisms and Implications with Visceral Adipose Tissue

**Figure 2:**
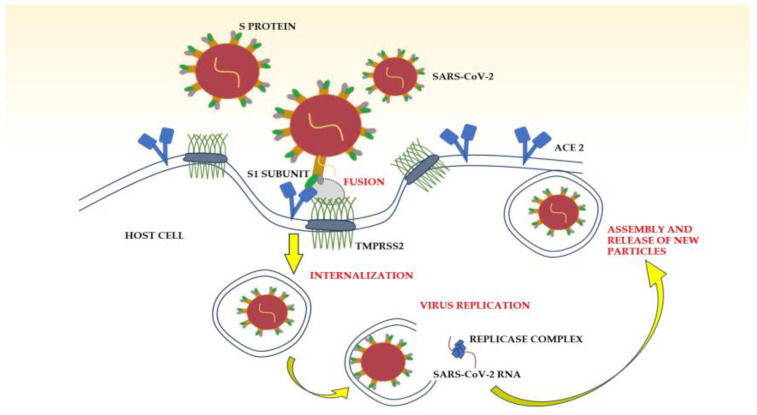
Pathogenic mechanisms of SARS-CoV-2 infection. ACE2: angiotensin-converting enzyme 2; S Protein: spike protein; SARS-CoV-2: severe acute respiratory syndrome coronavirus 2; TMPRSS2: transmembrane protease serine 2.

SARS-CoV-2 is a single-strand, positive-sense RNA genome virus, belonging to the Coronaviridae family; it is characterized by an envelope, on the surface of which are located projections known as spikes [19]. The genomic organization of the virus shows sequential arrangement of structural, non-structural and accessory genes to encode and synthesize two groups of protein. Structural proteins, namely spike (S), membrane (M), envelope (E) and nucleocapsid (N) proteins, are important to virus assembly, release and binding of host cells. In particular, spike protein consists of S1 and S2 subunits: the interaction between the S1 subunit and angiotensin-converting enzyme-2 (ACE2) receptors grants entry of the virus into the host cells. This process is crucial, as S-protein-RNA-based vaccines have been developed against SARS-CoV-2. The overall mechanism of interaction between the virus and the host has been fully explained. As previously stated, SARS-CoV-2 enters the target cell, binding to the receptor protein ACE2, which is located in many human tissues, such as the lungs, heart and adipose tissue [20,21]. When the S1 subunit of the spike protein interacts with the receptor, it is cleaved by transmembrane serine protease 2 (TMPRSS2), a serine protease expressed by the host cell, to allow fusion of the virus with the plasma membrane [22]. Subsequently, the virus starts its replication inside the host cell, producing structural and non-structural proteins, which are combined with RNA to create copies of the virus that are able to infect other host cells (Figure 2).

The aim of this review is to focus on the risk of poor outcomes of COVID-19 in patients with obesity; in particular, VAT accumulation is considered a negative prognostic factor in COVID-19 patients, rather than body mass index (BMI) [23], as shown by meta-analysis of epidemiological studies. COVID-19 patients requiring intensive care unit (ICU) admission or invasive mechanical ventilation (IMV) had greater VAT depots than patients who did not [24]. However, it is notable that excess subcutaneous adipose tissue is not associated with a higher risk of severe forms of COVID-19 [25]. It was also reported that epicardial adipose tissue (EAT), quantified using chest CT, is independently associated with the extent of pneumonia and adverse outcomes in patients with COVID-19, lending support to the use of EAT in clinical risk stratification [26]. Furthermore, anthropometric indicators of obesity, for instance, waist circumference and waist-to-hip ratio, are correlated with a higher incidence of COVID-19 mortality [27]. These observations suggest body composition and anthropometric indicators, along with BMI, as predictors of the potential risk of severe forms of COVID-19. The severity of COVID-19 is determined by the initial viral load of SARS-CoV-2, such as that seen in patients with severe forms compared to non-severe forms. High viral load (more than 10^9^ copies) of SARS-CoV-2 in elderly patients (older than 70 years old) at an early stage of the disease results in a poor outcome [28]. Magleby et al. evaluated the viral load in hospitalized patients with COVID-19 and observed that high viral load among those patients independently correlated with the risk of intubation and in-hospital mortality [29]. Importantly, plasma viral load has been associated with other indicators of severity, such as inflammatory mediators and IL-6 [30], which are typically elevated in patients with obesity, promoting the cytokine storm.

Obesity is characterized by hypertrophic expansion of adipose tissue due to augmented storage of lipids. It has been shown that mature adipocytes overexpress ACE2 [31], determined by the activation of the transcription factor PPARγ. Interestingly, the expression of ACE2 is higher in visceral adipose tissue, rather than subcutaneous adipose tissue [32]; VAT is located around several organs, where it takes the name of intrathoracic fat, epicardial fat or mesenteric fat, and a recent study demonstrated that ACE2 gene expression is higher in visceral and subcutaneous adipose tissues than in lung tissue, which is the major target tissue of SARS-CoV-2 [33]. Furthermore, ACE2 expression is enhanced by several proinflammatory cytokines [34], the levels of which are already elevated in obese patients. In turn, the elevated expression of ACE2 in the adipose tissue of obese patients may determine greater viral entry and replication [35], suggesting a role for adipose tissue as a virus reservoir, enhancing viral spread [36]. The function of adipose tissue as a virus reservoir has been previously reported to be a factor in the persistence of other viruses [37]. For this reason, VAT is considered a good target for SARS-CoV-2 infection, and its enlargement explains why obese patients have a greater risk of infection and adverse outcomes. It is necessary to emphasize that visceral adiposity, particularly EAT, has been linked to adverse outcomes and poor prognosis in COVID-19. The reason for EAT’s role in worse COVID-19 outcomes is related to its thoracic localization, directly influencing the cardiovascular complications of COVID-19 [38,39]. A Mexican study [40] documented that EAT thickness was associated with increased COVID-19 mortality regardless of age, gender, comorbid conditions and BMI. In detail, EAT was associated with lower SpO2 and PaO_2_/FiO_2_ indices and higher levels of cardiac troponins, D-dimer, fibrinogen and C-reactive protein and higher 4 C severity scores [40].

## 4. Influences of the Adipose Tissue on the Clinical Behavior of COVID-19

### 4.1. Emerging Data about COVID-19 in Obese Subjects

**Figure 3:**
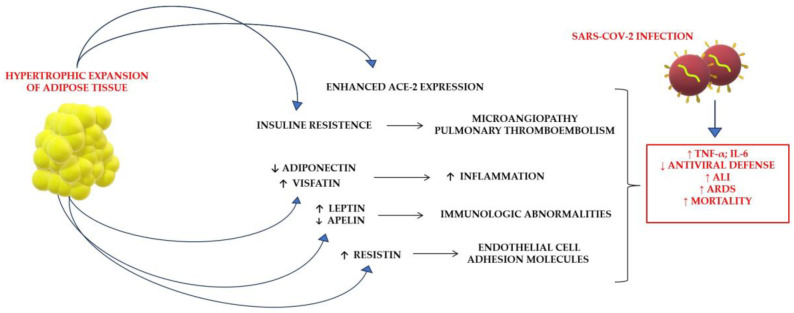
Postulated implications of obesity during SARS-CoV-2 infection. ACE-2: angiotensin-converting enzyme 2; ALI: acute lung injury; ARDS: acute respiratory distress syndrome; IL-6: interleukin 6; TNF-α: tumor necrosis factor alpha. Arrows indicate induced responses.

Obesity is a chronic metabolic condition characterized by altered systemic metabolism, including insulin resistance, increased lipid concentrations, abnormal body fat storage, altered adipokine profiles (e.g., increased leptin and decreased adiponectin) and chronic low-grade inflammation. BMI represents the most rapidly available anthropometric parameter to predict overweight and obesity status. At the same time, it is of paramount importance to recognize that BMI is an indirect measure of body fat, especially compared with direct approaches, such as bioelectrical impedance [41,42,43,44]. Several systematic reviews and meta-analyses have demonstrated that obesity represents a fundamental risk factor for development of and the prognosis for COVID-19 disease. However, regarding the risk of developing the disease, controversies persist among authors, some of whom reported an increased risk of developing COVID-19 of up to 46% and others who reported no increased risk [45,46,47]. Regarding the prognosis, it has been largely found that obese COVID-19 patients have worse courses of the disease in terms of hospitalization (113%), respiratory failure, ICU admission (74%) and mortality (48%) [46,48]. However, since many authors have outlined the possibility that BMI and overweight/obesity may represent confounding factors rather than risk factors for the poor prognosis of COVID-19, most studies have ruled out this possibility by applying statistical assays. A large population-based study, which avoided the risk of collider bias, found that a BMI of 30 kg/m^2^ or greater was associated with a slightly higher risk of death from COVID-19 compared to having a BMI less than 30 kg/m^2^. The mechanisms underlying the severe predisposition that obesity represents in COVID-19 are complex and not yet fully understood. It is well known that obesity, characterized by abnormal body fat content, is associated with several complications, including hypertension, dyslipidemia and type 2 diabetes (T2D). The last represents one of the key molecular features linking obesity to poor prognosis of COVID-19. Indeed, the production of oxidants and glycation products can directly damage immune function and induce inflammation through the secretion of effector cytokines, such as tumor necrosis factor alpha (TNF-α) and interferon-gamma (INF-γ), which in turn drive the imbalance in pro-inflammatory immune cells, such as Th1 cells, macrophages and dendritic cells. Along with this immune dysregulation, hypertrophic adipocytes contribute to the creation of an inflammatory environment that secretes cytokines, such as IL-6 and adipokines. In obesity, adipose tissue undergoes a modification involving an increase in the size and number of adipocytes and infiltration of immune cells. The chronic low-grade inflammatory environment typical of obesity, in which circulating cytokines, including IL-6, are hyperactivated, further contributes to immunosuppression and disproportionate cytokine activation. Furthermore, dysfunctional regulation of adipokines has also been implicated in the development of inflammatory and immune disorders (Figure 3).

### 4.2. Adipose Tissue and Vascular Endothelium Dysfunction

One of the most important acute complications in severe cases of COVID-19 is microangiopathy, often leading to pulmonary thromboembolism [49]. A recent metanalysis found the incidence of pulmonary embolism to be 21%. In addition, the incidence of pulmonary embolism in ICU patients with COVID-19 was higher than that in non-ICU patients with COVID-19 [50]. Adipose tissue accumulation is a risk factor for thrombotic complications, which can contribute to adverse outcomes in patients with COVID-19. These patients may have a higher risk of pulmonary thromboembolism compared to those with normal BMI. Diffuse endothelial inflammation and a pro-coagulant state have suggested that the vascular endothelium represents a target for SARS-CoV-2 and could cause impaired microcirculation [51,52,53]. In obese patients there is a pro-thrombotic state, which explains the greater risk of thrombotic complications, which is linked to the decrease in fibrinolytic activity and increased platelet activation [54]. Aljada et al. [55] suggested that the reduction in fibrinolytic activity is a consequence of the endocrine alterations associated with obesity and, in particular, with insulin resistance: insulin influences the level of plasminogen activation inhibitor-1 (PAI-1) by regulating the transcription of EGR-1.

Furthermore, insulin-induced anti-aggregation observed in subjects with normal BMI is blocked in patients with obesity-related insulin resistance, indicating that obesity is closely related to platelet activity abnormalities [56]. In fact, obese subjects present greater platelet activation due to increased excretion of 11-dehydro TBX2, a metabolite of thromboxane [57]. All these findings show that there is a hypercoagulable state in obesity, and it is one of the factors contributing to the higher risk of thrombotic complications in COVID-19 patients with obesity [44].

### 4.3. Dysregulation of the Renin–Angiotensin System and COVID-19-Related Respiratory Dysfunction

As noted above, impaired respiratory function progressing to ARDS is the most common complication of COVID-19. Obesity increases the risk of developing respiratory dysfunction, not only with mechanical effects but also by molecular pathways involving the renin–angiotensin system (RAS), which is crucial for blood pressure regulation and electrolyte balance and affects the functions of different organs, including the heart, lungs and kidneys [58]. Since ACE2 expressed on the adipocytes of obese patients and is a target molecule for SARS-CoV-2, this interaction leads to hyperactivation of the RAS pathway, which has been investigated as one of the potential mechanisms contributing to the worse prognosis of COVID-19 subjects with concomitant obesity.

COVID-19 patients with obesity have a higher risk of thrombotic complications [44].

The binding of SARS-CoV-2 to the target cells through the viral spike protein, followed by entry into the cell, is associated with a decrease in ACE2 [59]: a decrease in ACE2 may alter the balance between pro-inflammatory angiotensin-II (Ang-II) and anti-inflammatory angiotensin (1–7). Indeed, serum Ang-II levels have been shown to be elevated in COVID-19 patients [60], indicating reduced action of ACE2. Accumulation of Ang-II causes pulmonary dysfunction, playing a vital role in the stimulation of lung permeability, causing pulmonary edema and inflammation [61].

## 5. General Characteristics and Functions of Adipokines and Their Involvement in COVID-19: Leptin, Adiponectin, Resistin, Apelin, Visfatin and Omentin

Adiponectin: Adiponectin, also referred to as ACRP30, AdipoQ and gelatin-binding protein-28, a polypeptide of 244 amino acids, is the most abundant adipokine synthesized and released from adipose tissue [59,60] and circulating as an oligomer of different molecular weight: trimers (LMW), hexamers (MMW) and high molecular weight oligomers (HMW) [61]. The last are the most relevant oligomers in terms of biological activities. Often measured in studies involving obese subjects, this cytokine has been inversely linked to BMI, body fat content and inflammation [62]. Notably, hypoadiponectinemia is reported in obesity and obesity-linked complications, including type 2 diabetes, and coronary heart disease and hypertension [63]. Beyond favorable metabolic actions, so far, adiponectin has been investigated for its potential associations with inflammatory states; literature from the data have reported a duality in adiponectin functions (i.e., with both pro-inflammatory and anti-inflammatory effects) contributing to the pathogenesis of several diseases.

However, overall, the published studies concur in defining an inverse relationship between adiponectin levels and several pro-inflammatory markers in various patients, concluding that this adipokine predominantly possesses anti-inflammatory functions [63]. Mechanistically, adiponectin acts on immune cells, as well as epithelial and vascular cells, inhibiting the release of pro-inflammatory regulating proteins.

In light of its anti-inflammatory role and with regard to COVID-19, adiponectin expression has been widely investigated (Table 1). A decrease in plasma levels of adiponectin was observed in COVID-19 patients with respiratory failure compared to non-COVID-19 patients also experiencing respiratory failure [64]. Recently, we investigated whether adiponectin and HMW oligomer serum levels were modulated and/or correlated with clinical and biochemical parameters of severe COVID-19 patients, demonstrating that HMW oligomers might represent a laboratory resource to predict patient clinical outcomes [65]. Accordingly, Flikweert et al. [50] showed that lower adiponectin levels are found in severe and critical patients, compared to those not necessitating hospitalization. Such associations between adiponectin and worse prognosis are probably related to the loss of adiponectin functions in terms of anti-inflammatory properties; in turn, the worsening of inflammation, already compromised by SARS-CoV-2 infection, leads to respiratory failure. [66]. Ultimately, most of the literature agrees in defining a clear role for adiponectin in contributing to adverse outcomes in COVID-19 patients [65].

Leptin: Leptin is mainly secreted by white adipose tissue, with its levels positively correlated with the amount of body fat; initially noted for its role in the regulation of energy homeostasis and metabolism, leptin exerts a plethora of other effects, such as regulation of immune function and inflammation [67].

Hyperleptinemia has been described as one of the clinical parameters associated with SARS-CoV-2 infection [68,69] (Table 1). Van de Voort et al. reported higher levels of serum leptin in severe SARS-CoV-2 patients compared to mild cases [51]. Mechanistically, leptin levels have been associated with the immunologic aberrations and systemic pro-inflammatory state that typically occur in COVID-19 patients [51,67]. In addition, Baltodano-Calle et al. suggested leptin as a biomarker of the inflammatory state, possibly suitable for determining the risk of COVID-19 and its possible clinical complications [70]. The molecular cause of the hyperleptinemia in COVID-19 has been hypothesized to be linked to the expansion of the cytokine storm related to disease exacerbation (acute respiratory distress syndrome and multiple organ failure), especially in obese patients [67]. Tonon et al., in a very recent paper, analyzed patients with COVID-19 pneumonia, finding that leptin and the adiponectin/leptin ratio had adequate discriminatory accuracy for COVID-19 pneumonia [71]. Similarly, di Filippo et al. found that high adiponectin/leptin ratios are related to inflammation in COVID-19 [72]. It is notably that most of the patients analyzed by Tonon et al. were severely obese (BMI > 30) while Di Filippo et al. considered mainly mild to moderate cases [71,72].

Resistin: Resistin, first discovered in mice in 2001, is named for its ability to resist insulin action [73]. In humans, it is mainly secreted by macrophages and also by adipocytes. Such specific production by immune cells paved the way for subsequent investigations that outlined resistin regulation and interference with inflammatory responses; such functions are performed mainly through the promotion of the release of pro-inflammatory cytokines in both macrophages and endothelial cells [74]. Although initially described as a detrimental factor in the pathogenesis of insulin resistance and type 2 diabetes, resistin has since been implicated in inflammatory diseases [75].

Our published data [65], in accordance with few available data in the literature, showed that resistin is higher in COVID-19 patients and has been found to predict the requirement for invasive ventilation [76]. A very recent paper showed that elevated resistin levels are associated with cytokines and endothelial cell adhesion molecules and are related to COVID-19 disease severity scores and worse outcomes [77] (Table 1).

Apelin: Apelin is a neuropeptide produced as a precursor of 77-amino acid named preproapelin, which, after clivation at the C-terminus, yields the following active peptides: apelin-13, -16, -17, -19 and -36 [78]. The isoform -13 is the most biologically potent form, while all peptides are widely expressed in various human tissue types, predominantly in adipose tissue [79]. The functions of apelin are mediated by binding to a G-protein-coupled receptor called APJ [80]. The APJ receptor, located in the endothelium, smooth muscle cells and cardiomyocytes, is a G-protein-coupled transmembrane receptor, sharing more than 50% amino acid homology in transmembrane regions with angiotensin receptor 1 (AT-1) [81]. The functional significance of apelin down-regulation has not been fully elucidated, but it has been suggested that apelin may ameliorate Ang-II-mediated inflammation, thrombosis and vasoconstriction in COVID-19.

Serum levels of apelin are lower in COVID-19 patients with concomitant hypertension or in patients with concomitant diabetes mellitus and obesity, making them vulnerable to a more severe disease form [82]. Serum apelin has also been proposed as a biochemical factor predicting mortality in severe COVID-19 patients [83].

Mechanistically, a recent in vitro study demonstrated that apelin-13 inhibits cell-to-cell fusion mediated by ACE2 binding to the S protein; in addition, apelin acts by controlling inflammatory responses to viral infection by inhibiting the nuclear factor kappa B pathway [84].

Together, these data suggest that apelin or its receptor agonists could be of major interest in developing a potential treatment for COVID-19 through ACE and Ang-II suppression, as well as down-regulation of angiotensin receptor 1 (AT1R) and up-regulation of ACE2 [85] (Table 1).

Visfatin: Visfatin/nicotinamide phosphoribosyltransferase (NAMPT) is an adipocytokine predominantly produced by visceral adipose tissue. It is also expressed in the bone marrow, liver, muscles, heart, placenta, lungs and kidneys [86,87,88]. The homodimeric protein possesses phosphoribosyltransferase activity [89], functioning as an immune modulatory cytokine capable of inducing pro-inflammatory responses through the release of many inflammatory mediators [90,91]. Therefore, the protein has aroused great interest in reference to inflammatory disorders; the literature clearly shows that visfatin secretion is increased in many inflammatory diseases, including lung disorders [92]. From a molecular point of view, visfatin can stimulate the release of pro-inflammatory cytokines in different cell types, including immune cells (monocytes, neutrophils, B cells) and endothelial cells [93].

Few data are available on visfatin expression in COVID-19 (Table 1). In a large cohort study of 260 SARS-CoV-2-infected individuals with varying degrees of severity [94], visfatin levels were higher in critically ill patients with COVID-19 than in non-COVID-19 mild and severe ICU patients. Lower adiponectin levels but higher resistin levels were found in the same severely and critically ill patients, compared to those who did not require hospitalization. The authors concluded that circulating adipokine levels are associated with hospitalization for COVID-19 but not with mortality.

Omentin: Also known as intelectin, intestinal lactoferrin receptor and endothelial lectin HL-1, omentin is an adipokine expressed mainly in the stromal vascular fraction cells of adipose tissue [95] and circulating in human blood [96]. From a mechanistic point of view, omentin is implicated in metabolic functions (energy homeostasis, glucose metabolism) and in additional processes, such as inflammatory responses and oxidative stress [97].

To our knowledge, only one publication has analyzed omentin in COVID-19 patients. Kukla et al. measured the concentrations of chemerin, omentin and vaspin in serum from 70 COVID-19 patients compared to 20 healthy controls. Serum concentrations of chemerin and omentin were significantly reduced in COVID-19 patients compared to healthy volunteers, although no correlation was found with severity of the disease [98] (Table 1).

**Table 1:** Most recent and relevant studies exploring the role of adipocytokines in COVID-19.

Adipocytokine	Study Type	Study Population	Main Findings	Reference
Adiponectin	1. Retrospective	1. 12 COVID-19 patients vs 17 patients with other respiratory infections	1. Reduced adiponectin levels in acute respiratory distress in COVID-19 vs non-COVID-19	1. [66]
	2. Retrospective	2. 62 hospitalized COVID-19 patients vs 62 controls	2. Negative correlation of HMW adiponectin with LUS scores	2. [65]
	3. Multi-center, prospective, cross-sectional study	3. mild, severe, and critical COVID-19 vs non-COVID-19	3. Lower adiponectin levels in severe and critical patients compared to those who did not require hospitalization.	3. [50]
	4.Retrospective	4. 123 COVID-19 patients	4. Association of a doubling of circulating adiponectin with a reduction in 90-day mortality and respiratory failure	4. [69]
Leptin	1. Cross-sectional study	1. 31 SARS-CoV-2 patients requiring mechanical ventilation vs 8 patients with etiologies not related to SARS-CoV-2 infection	1. Increased leptin levels in COVID-19 associated with severity	1. [51]
	2. Retrospective,	2. 31 COVID-19 patients, 67.8% mild	2. Higher leptin levels in COVID-19 patients with activation of monocytes	2. [99]
	3. observational, case-control study	3. 48 COVID-19 patients and 36 healthy controls	3. Higher levels of leptin, lower adiponectin/leptin (Adpn/Lep) ratio	3. [71]
	4. Single-center, prospective, observational study	4. 60 patients with mild, moderate and severe COVID-19	4. Patients with moderate severity had the highest Adpn/Lep ratios, correlated with systemic inflammation	4. [72]
Resistin	1. Retrospective	1. 62 hospitalized COVID-19 patients vs 62 controls	1. Increased resistin levels in COVID-19 patients compared to controls. Resistin levels predicted COVID-19 severity and need for respiratory support	1. [65]
	2. Retrospective	2. 306 COVID-19 patients and 38 sepsis patients	2. Elevated resistin in COVID-19 patients related to worse outcomes and to cytokine and endothelial cell adhesion molecules	2. [76]
	3. Observational	3. 195 hospitalized COVID-19 patients.	3. Resistin correlation with inflammatory markers and higher in ICU patients and non-survivors	3. [77]
Apelin	Case–control study	69 COVID-19 patients and 71-matched non-COVID-19 participants	Lower apelin content in the COVID-19 and COVID-19 + diabetes mellitus groups compared to the non-COVID-19 counterpart groups. Positive association with arterial SO2 and negative association with the severity of lung involvement	[82]
Visfatin	Multi-center, prospective, cross-sectional study	260 mild, severe and critical COVID-19 patients	Increased visfatin levels across different COVID-19 stages. Critical COVID-19 visfatin levels were higher compared to those in critical non COVID-19 patients	[50]
Omentin-1	Retrospective	70 COVID-19 patients	Reduced omentin-1 levels were observed in COVID-19 patients compared with controls. No significant association with COVID-19 severity were observed.	[98]

## 6. Adipokines Modulate Inflammatory Cytokines Expression in COVID-19

As mentioned above, plasma levels of adipokines and cytokines are strictly associated with the establishment and severity of COVID-19. The inflammatory response to SARS-CoV-2 infection plays an important role in the progression to severe COVID-19 disease. In a recent study, Flikweert et al. [50] observed that, in severely ill patients, IL-6 levels were positively correlated with the levels of adiponectin, visfatin and resistin and negatively correlated with plasma leptin levels. Van de Voort et al. also found that patients with severe COVID-19 had higher levels of serum leptin than those with mild disease [51].

Interestingly, leptin and adiponectin exert opposite effects on metabolism, immunity and inflammation, and obese individuals have an inverse expression pattern: leptin is increased, while adiponectin is down-regulated. Therefore, the ratio of these two adipokines in circulation might represent a relevant biomarker to be considered in the pathophysiology of COVID-19 [52].

The worsening clinical state in COVID-19 is related to the elevation of pro-inflammatory cytokines, such as TNF-α and IL-6, in a condition known as cytokine storm. The two main complications, which carry a higher risk of mortality, that result from the cytokine storm are ARDS and pulmonary embolism. Since TNF-α plays an important role in promoting ARDS, obese patients, who are already characterized by elevated TNF-α levels, have higher risk of developing a more severe COVID-19 phenotype [54]. Similarly, IL-6 levels are significantly elevated and associated with adverse clinical outcomes of COVID-19. Indeed, circulating levels of IL-6 are higher in hospitalized COVID-19 patients with complications compared to patients without complications [55].

Furthermore, obese patients are more vulnerable to unfavorable outcomes in SARS-CoV-2 infection because the damaged metabolic and endocrine functions of AT, as well as the general chronic inflammatory state, affect the normal functions of key organs, such as the lungs, kidneys and heart. In particular, high leptin levels and adiponectin reduction lead to oxidant imbalance, reduced immune response and endothelial dysfunction: as the virus spreads throughout the organism, cytokine storm is promoted, and direct lung inflammation develops into clinical forms of ARDS and acute lung injury (ALI), which are common in severe cases of COVID-19. The role of adipocytokines in severe cases has been extensively evaluated. Low levels of adiponectin, typical in obese patients, predispose these patients to ALI by promoting inflammatory responses in the pulmonary vascular endothelium [100]. Furthermore, adiponectin has been shown to protect rats from ALI by acting as an endoplasmic reticulum (ER) stress inhibitor [101] through the activation of specific molecular pathways, such as PPAR-α and AMPK [102]. In this context, the combination of hypoadiponectinemia and ER stress promoted by SARS-CoV-2 infection further increases the risk of ALI and, consequently, adverse outcomes in obese patients. Leptin plays a role in the adverse outcomes of COVID-19: elevated circulating levels of leptin in patients with obesity, associated with the secretion of other cytokines, impair host antiviral defense and reduce the immune response against the virus, leading to clinical aggravation, ARDS and multiple organ failure. Elevated leptin levels by bronchoalveolar lavage (BAL) in patients with diabetes and ARDS are associated with increased mortality [103]. Gattinoni et al. [104] observed that COVID-19 does not lead to a “typical” ARDS, as they described a form with severe hypoxemia but preserving lung mechanics. In patients with obesity, VAT excess in the thoracic and abdominal areas compromises the mechanics of the chest wall, reducing the functional residual capacity: in these patients, pleural pressure is increased, and the excursion of the diaphragm is limited [105], making ventilation difficult, especially in the supine position [106]. The mechanical effects of lung compression led to worsening respiratory symptoms and clinical outcomes in obese patients with COVID-19. The metabolic alterations of obesity cooperate to impair host antiviral defenses against SARS-CoV-2 infection, explaining the high mortality rates in individuals with concomitant obesity [107].

## 7. Conclusions

The role of adipose tissues as an endocrine gland secreting hormone-like substances known as adipokines outlines a possible determinant role in physiology and pathophysiology of metabolic and non-metabolic disorders due to the fundamental role that the adipokines plays in processes such as inflammation and immunity, both of which are involved in the onset and establishment of COVID-19. Furthermore, by contributing to regulation of the inflammatory response, adipokines are closely related to the prognosis of the disease; the situation is even more complicated in patients affected by concomitant metabolic disorders.

Indeed, altered secretion of adipokines in COVID-19 patients with obesity typical of excessive and dysfunctional adipose tissue may amplify systemic inflammation in such patients, worsening the prognosis. Among the adipokines, adiponectin appears to be the most promising hormone. Its down-regulation is clearly demonstrated, with a concrete possibility of determining the dosage for diagnostic purposes to identify patients with poor prognosis. The functional interference of adiponectin in the inflammatory environment established by SARS-CoV-2 infection is paving the way for further experimental evaluations that will clarify the potential of adiponectin as a therapeutic target in COVID.

## Data Availability

Not applicable.
